# Isolation Frequency of Fluconazole-Resistant Candida Species From Cockroaches: A Cross-Sectional Study From a National Hospital in Dar es Salaam, Tanzania

**DOI:** 10.7759/cureus.24412

**Published:** 2022-04-23

**Authors:** Doreen Mloka, Raphael Z Sangeda, George M Bwire, Kennedy D Mwambete

**Affiliations:** 1 Department of Pharmaceutical Microbiology, Muhimbili University of Health and Allied Sciences, Dar es Salaam, TZA

**Keywords:** candida glabrata, hai, hospital-acquired infections, aspergillus fumigatus, aspergillus niger, candida albicans, fluconazole resistance, fungi, cockroaches

## Abstract

Background: Cockroaches are common pests in homes and hospitals. They cause allergic reactions in some individuals and are potential vectors for various infectious pathogens. The study investigated the extent to which hospital cockroaches act as vectors and reservoirs of medically important fungal pathogens on their external surfaces.

Methods: Cockroaches were captured from the selected hospital locations including the burn unit, adult surgical wards, pediatric oncology wards, intern hostel kitchen, and the central kitchen of a national referral teaching hospital in Tanzania. Normal saline washings from the external surface of cockroaches were cultured on standard mycological media to facilitate isolation and identification of medically important molds and yeasts. The susceptibility of *Candida* species isolates to fluconazole was tested using the Clinical and Laboratory Standards Institute (CLSI) M27-A3 microdilution method.

Results: A total of 69 cockroaches were captured from various hospital sites between February and April 2017. All cockroaches captured were shown to carry medically important fungi. A total of 956 medically important fungi were isolated; 554 (57.9%) were of *Candida* species, 222 (23.2%) were of *Aspergillus* species, 30 (3.1%) were of​​​​​​​ *Cladosporium* species, 17 (1.8%) were of​​​​​​​ *Rhizopus* species, 11 (1.2%) were of​​​​​​​ *Geotrichum* species, nine (0.9%) were of​​​​​​​ *Penicillium* species, seven (0.7%) were of​​​​​​​ *Alternaria* species, six (0.6%) were of​​​​​​​ *Fusarium* species, three (0.3%) were of​​​​​​​ *Mucor* species, and 97 (10.1%) were of other species.* *Of the *Aspergillus* species,* Aspergillus fumigatus* (111, 50.0%) was the most commonly isolated, followed by *Aspergillus niger* (35, 15.8%) among the *Aspergillus* isolates. Out of the 103 selected isolates, 18 (17.5%) of the *Candida* isolates normally not intrinsically resistant to fluconazole demonstrated resistance to this drug. Resistance was most frequently found in *Candida parapsilosis* (3, 30%), *Candida pseudotropicalis* (10, 23.8%), and *Candida glabrata* (2, 18.2%). The isolates with the least proportion of resistance to fluconazole were *Candida albicans* (2, 6.3%).

Conclusion: Cockroaches from this hospital may act as reservoirs of medically important opportunistic fungi exhibiting resistance to fluconazole.

## Introduction

Cockroaches belong to the order Orthoptera or Dictyoptera and the families Blattidae or Blattellidae. They are common pests in domestic dwellings, hospitals, and industrial areas. They tend to live hidden in the dark cracks and crevices of kitchens, toilets, and food stores, which are ideal environments due to temperature, humidity, and sources of nutrition for them to flourish. Kitchens and toilets are areas that are often contaminated with infectious microorganisms, including bacteria, viruses, protozoa, and fungi. Therefore, cockroaches in these areas may likely be reservoirs of infectious pathogenic microorganisms.

Healthcare-associated infections (HAIs) are a major public health concern worldwide because they contribute to increased morbidity, mortality, and cost [1,2]. The WHO estimates that the prevalence of HAIs varies between 5.7% and 20.0% in low- and middle-income countries (LMICs) [1]. Several studies have suggested that hospital cockroaches may be potential carriers of infectious microorganisms, including drug-resistant bacteria [2-8]. Nevertheless, limited studies have looked at the role of cockroaches as vectors and reservoirs of opportunistic and HAI associated with fungal pathogens [9]. Although eliminating HAIs is impossible, regular surveillance and effective infection control prevention (ICP) programs can help reduce its incidence. However, its implementation in LMICs is challenging. Many hospitals in LMICs often lack adequate staff, equipment, and supplies to institute ICP measures effectively. Basic ICP practices such as hand hygiene are usually not adhered to the lack of clean running water or lack of budget for sanitizing agents [3]. Moreover, the inability to regularly monitor and evaluate HAIs in LMICs may delay detecting outbreaks, resulting in increased morbidity, mortality, and cost associated with these infections [4-6].

Hospital environments provide the perfect environment in terms of temperature, humidity, and sources of nutrition for them to flourish. Despite these impediments, it is imperative that healthcare institutions, especially in LMICs, strive to identify HAI sources and implement preventative measures to reduce the incidence of HAIs in their environments. There are limited published data on the prevalence of HAIs. In particular, data on the non-fomite vectors of fungal HAIs are lacking [7-10]. Therefore, we conducted this study to exemplify the role of cockroaches as vectors and reservoirs of HAI-associated fungal infections in Tanzania.

## Materials and methods

Settings

The study to determine the prevalence of medically important fungi on the surface of hospital cockroaches was conducted at a national referral teaching hospital in Dar es Salaam, Tanzania. The facility is a 1,500-bed hospital, attending 1,000-1,200 outpatients per day, and admitting 1,000-1,200 inpatients per week.

Study design

A cross-sectional study was conducted where hospital cockroaches were trapped over two months between February and April 2017. Cockroach adhesive sticky paper was placed overnight on the floors of wards of the burn unit, intensive care unit (ICU), surgical department, neonatal unit, hospital's main kitchen, pediatric oncology, and the students' intern hostel kitchen. Trapped live cockroaches were collected using sterile forceps and placed in sterile capped test tubes for transportation to the laboratory immediately for processing.

Fungal isolation, culture, and identification

Sterile normal saline (10 ml) was added to each test tube and the live cockroaches were thoroughly shaken for two minutes. Cockroach washings (5 ml) were then aseptically removed to a sterile tube for six sets of 10 serial dilutions ranging from 1/10 to 1/10^6^ and were then serially diluted in sterile water; 1 ml duplicate aliquots of the washings were cultured on Sabouraud dextrose agar containing 0.5% chloramphenicol and 0.05% gentamicin. Plates were incubated at 30°C for three weeks and examined daily for viable counts. Filamentous fungi and yeast colonies were identified using their microscopic and macroscopic characteristics, such as topography, texture, pigmentation, mycelium type, hyphae form, spore type, and type of reproductive structure. Yeast was further identified by the germ tube test, the presence of chlamydoconidia on cornmeal plus Tween 80 agar (Oxoid, Hampshire, UK), and the color of colonies on CHROMagar™ Candida (Paris, France).

Antifungal susceptibility profile of fungal isolates

Susceptibility to fluconazole on 103 randomly selected *Candida* species isolates was tested using the Clinical and Laboratory Standards Institute (CLSI) M27-A3 microdilution method [11]. Reference grade fluconazole (Sigma-Aldrich, St. Louis, MO) was obtained in powder form. Antifungal susceptibility testing was performed in microdilution plates in which 0.1 mL of fluconazole three-times concentrate was used. The inocula were prepared from overnight (24-48 hours) cultures in Sabouraud dextrose agar. The inoculum suspension was prepared using a spectrophotometer to produce a 0.5 McFarland standard at 530 nm wavelength to produce standard inoculum of approximately 1 x 10^6^ cells per ml. The stock fluconazole solution was diluted in RPMI-1640 (Sigma-Aldrich, St. Louis, MO) buffered with morpholinepropanesulfonic acid (MOPS). Freshly prepared microdilution plates containing 100 µL of RPMI-1640 and serial dilutions of fluconazole were inoculated with 100 µL of diluted culture, resulting in 0.5 x 10^3^ to 2.5 x 10^3^ cells/mL in each well. The plates were incubated at 35°C for 24-48 hours, and the minimum inhibitory concentration (MIC) endpoint was determined and interpreted according to CLSI M27-A3 guidelines. *Candida parapsilosis *ATCC 22019 (Manassas, VA) served as the quality control isolate.

Data processing and analysis

Laboratory data were entered and analyzed using Statistical Package for the Social Sciences (SPSS) version 20 (IBM Corp., Armonk, NY). Descriptive statistics were presented as percentages in tables and figures.

## Results

**Figure 1:**
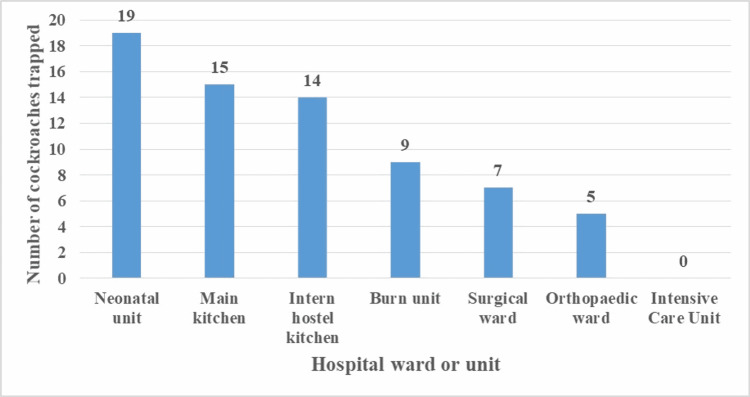
Number of cockroaches trapped per hospital ward/unit

A total of 69 cockroaches were captured from different wards of a tertiary teaching hospital (Figure 1).

The majority of cockroaches were trapped in the neonatal unit (19) followed by the interns' kitchen (14), burn unit (9), and main hospital kitchen (5). No cockroaches were captured from the main ICU.

**Figure 2:**
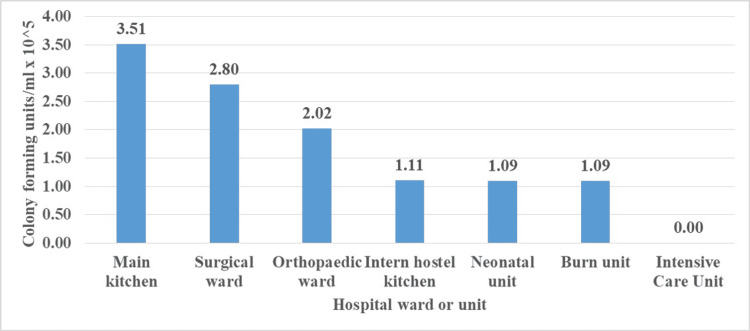
Fungal load in colony-forming units (CFUs) CFUs were measured as units per ml x 10^5^.

The highest total fungal viable counts were found in cockroaches from the main hospital kitchen (3.5 x 10^5^ cfu/ml) followed by the surgical wards (2.8 x 10^5^ cfu/ml) and pediatric oncology wards (2.0 x 10^5^ cfu/ml), respectively (Figure 2).

**Figure 3:**
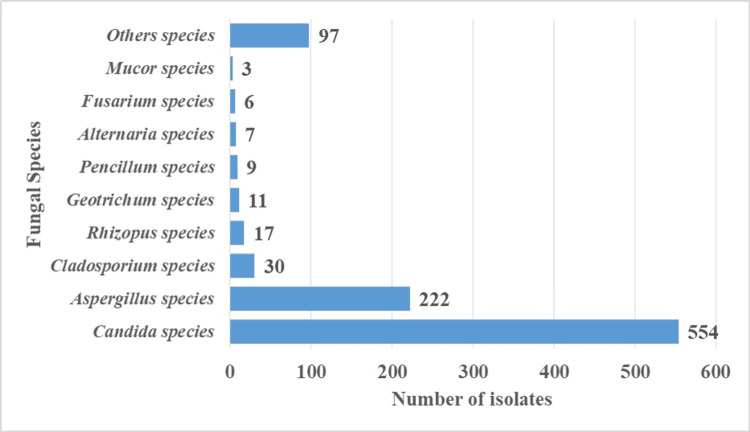
Number of fungal isolates from cockroaches’ surfaces

A total of 956 medically important fungi were isolated. The majority of isolates were of *Candida *species. Overall, 554 isolates were of *Candida *species, 222 were of *Aspergillus *species, 30 were of *Cladosporium *species, 17 were of *Rhizopus *species, 11 were of *Geotrichum *species, nine were of *Penicillium *species, seven were of *Alternaria *species, six were of *Fusarium *species, three were of *Mucor *species, and 97 were of other species (Figure 3).

Among the 103 randomly selected *Candida* isolates, 18 (17.5%) showed resistance to fluconazole. The majority of the resistant isolates were found in isolates of *C. parapsilosis*, *C. pseudotropicalis*, and *C. glabrata* (Table 1).

**Table 1:** Proportion of fungal species isolates resistant to fluconazole

Species	Number of isolates (N = 103)	Susceptible (S)	Susceptible dose-dependent (SDD)	R-resistant	Percentage resistant isolates (%)
C. pseudotropicalis	42	18	14	10	23.8
C. albicans	32	22	8	2	6.3
C. rugosa	8	6	1	1	12.5
C. parapsilosis	10	5	2	3	30.0
C. glabrata	11	8	1	2	18.2
Total	103	59	26	18	17.5

**Figure 4:**
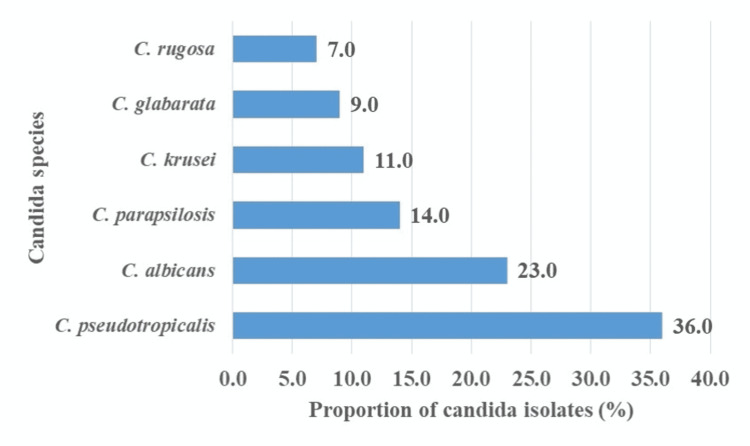
Candida species isolated on the external surfaces of cockroaches

Medically important *Candida *species, including* Candida albicans*, were isolated and identified (23%) (Figure 4).

**Figure 5:**
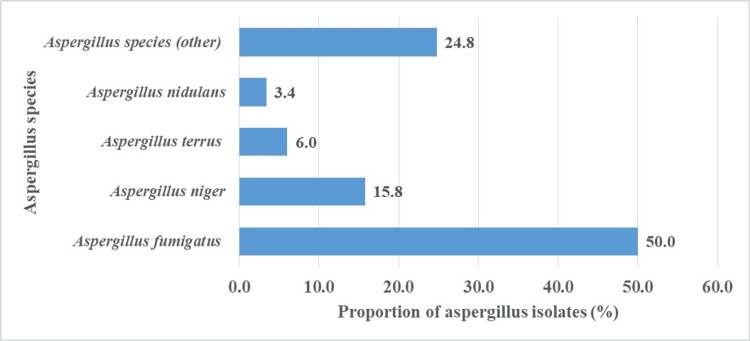
Proportion of Aspergillus species isolated on the external surfaces of cockroaches

In addition to *Candida* species, isolates of opportunistic pathogenic *Aspergillus *species like *Aspergillus fumigatus* (50%) were also identified (Figure 5).

## Discussion

Fungal HAIs continue to be a major contributor to increased morbidity, mortality, and cost of treatment, despite the current diagnostics and therapeutic options development. This study is the first description of the fungal profile of the external surfaces of hospital cockroaches in a Tanzanian tertiary teaching hospital. The study demonstrated that hospital cockroaches represent a potential reservoir of fungal pathogens associated with HAIs. The presence of seven medically important fungal genera, including *Candida*, *Aspergillus, Cladosporium*, *Mucor*, *Fusarium*, *Rhizopus*, and *Penicillium *on the outer surfaces of hospital cockroaches is similar to the results of other studies conducted in Iran, Iraq, and Brazil [12-14]. This high fungal carriage rate (100%) is significant in its implication regarding the transmission of fungal pathogens associated with HAIs, especially considering the habits of these insects that tend to move freely over various hospital areas. The fact that these insects, when feeding, regurgitate food from their crop and may defecate on hospital surfaces suggests that these insects may have a role to play in the spread of infectious fungal agents. The finding that a high percentage of tested cockroaches were contaminated with *Candida *species isolates (57.9%) and *Aspergillus *species (23.2%), respectively, seems further proof that these insects are indeed a vector for the spread of nosocomial fungal agents. Particularly considering the presence of *C. pseudotropicalis, C. albicans, C. parapsilosis*, *C. glabrata*, and *C. rugosa*, which are known fungal pathogens, i.e., increasingly being reported as agents of life-threatening and drug-resistant HAIs associated with high treatment costs, morbidity, and mortality [15]. *C. pseudotropicalis, C. albicans, C. parapsilosis*,* *and *C. glabrata* are important causes of bloodstream infection in neonates, transplant recipients, granulocytopenic patients, and surgical patients [15-20]. *C. rugosa* is increasingly recognized as an emerging pathogen of bloodstream infections in immunocompromised individuals [21].

The isolation of *Aspergillus fumigatus* and *Aspergillus niger* further supports the finding that hospital cockroaches may be potential reservoirs of major fungal pathogens associated with life-threatening HAIs of immunocompromised individuals. These isolates are major causes of invasive fungal infections, particularly in transplant and AIDS patients [18,19]. The presence of other known causes of opportunistic and nosocomial fungal infections species such as *Cladosporium*, *Mucor*, *Fusarium*, *Rhizopus*, and *Penicillium* further cements the concept that cockroaches in hospitals may be a major public health concern and confirms the findings of other studies conducted on domestic and hospital cockroaches [2,12-14,22-25]. The study findings highlight the need to conduct more studies to ascertain the medically important fungi on the surfaces of hospital cockroaches as possible etiological agents of HAIs including newer fungal pathogens in immunocompromised patients such as *Talaromyces marneffei* [26].

Recent study findings show that cockroaches in domestic and hospital settings are resistant to common pesticides [27]. The majority of cockroaches were trapped in the main hospital kitchen and no cockroaches were captured from the main ICU of this hospital. This indicates that infection prevention and control measures are being adhered to in sensitive hospital areas. However, there need to be concentrated efforts for effective fumigation practices in the food handling areas. Cockroaches carrying drug-resistant pathogens may wander from any part of the hospital through the hospital sewage systems unnoticed. Moreover, hospital environmental control teams, therefore, need to thoroughly assess the resistance of the hospital cockroaches to common pesticides before deciding what chemicals to use.

Fluconazole has been the cornerstone therapeutic option for many African countries in treating fungal infections, particularly vaginal and oral candidiasis. This study found that 16.3% of randomly selected *Candida *isolates exhibited resistance to fluconazole. This is higher than that previously reported in Tanzania, where it was shown that resistance to fluconazole was only 6.8% [27]. This finding may be a warning sign for the continued empirical use of fluconazole for prophylaxis and or treatment of systemic or localized *Candida *infections in African and Tanzanian healthcare settings, as resistance to fluconazole is on the rise [28,29]. The presence of fluconazole-resistant *Candida *species isolates on the outer surfaces of cockroaches thus clearly suggests that cockroaches can act as vectors to transmit drug-resistant fungal strains in hospital settings, especially within hospitals that lack effective pest control measures.

A limitation of this study is that it only determined the prevalence of fungal isolates on the surfaces of hospital cockroaches. The study did not use molecular tools to characterize the isolates and compare them with isolates of confirmed causes of HAIs at this hospital. Moreover, the antifungal susceptibility testing was only done in yeasts and used only one drug, i.e., fluconazole.

## Conclusions

Outer surfaces of hospital cockroaches may be an essential reservoir of potential drug-resistant fungal pathogens associated with HAIs. Hospitals need to assess their pest control measures to ensure they are effective against cockroaches. We recommend that similar studies should be done involving other potential infectious pathogen vectors commonly found in hospital environments, such as flies and rats.
